# Study on the Effects of Flow Fields Under the Refuge Effect of Artificial Reefs on the Behavior and Physiology of Juvenile *Lutjanus erythopterus*

**DOI:** 10.3390/biology15181660

**Published:** 2026-09-19

**Authors:** Yu Guo, Dezhong Kong, Changlin Li, Chuanxin Qin, Gang Yu, Zhenhua Ma

**Affiliations:** 1 South China Sea Fisheries Research Institute, Chinese Academy of Fishery Sciences, Guangzhou 510300, China; guoyu25895177@163.com (Y.G.); 15053442284@163.com (D.K.); lichanglin306@163.com (C.L.); gyu0928@163.com (G.Y.); zhenhua.ma@hotmail.com (Z.M.); 2 Key Laboratory of Efficient Utilization and Processing of Marine Fishery Resources of Hainan Province, Sanya Tropical Fisheries Research Institute, Sanya 572018, China

**Keywords:** *Lutjanus* *erythropterus*, flow field effect, artificial reefs, behavioral characteristics, physiological stress

## Abstract

Nearshore artificial facilities greatly alter coastal flow fields and habitats. As typical underwater artificial structures, offshore wind turbine pile foundations have unclear mechanisms for influencing fish habitats. Given the high similarity of flow disturbance and sheltering functions between wind pile foundations and artificial reefs, artificial reef models can be applied to analyze the ecological effects of pile foundations. Therefore, this study selected juvenile crimson snapper (*Lutjanus erythropterus*) as the research object and adopted cubic artificial reefs to simulate sheltered habitats so as to explore the behavioral and physiological responses of juvenile *L. erythropterus* under different flow-velocity conditions. The results indicated a significant positive correlation between flow velocity and oxygen consumption rate in the experimental area. Behavioral analysis showed that the time percentage spent stationary at 3 BL/s (63.23%) was significantly higher than that in the 1 BL/s (43.02%) and 5 BL/s (41.09%) groups. In contrast, the proportion of backward swimming time was highest at 5 BL/s and was comparable to the percentage of time spent stationary. Physiological analysis revealed that the GLU level in the 1 BL/s group was significantly higher than that in all other groups (*p* < 0.05). LD content peaked in the 5 BL/s group, where experimental fish also exhibited partial wall-hugging behavior and difficulty in controlling their swimming. There was no significant difference in LD levels between the 0 BL/s and 1 BL/s groups (*p* > 0.05). Additionally, as the flow velocity increased, the hepatic COR levels in *L. erythropterus* showed a corresponding upward trend, peaking at 5 BL/s. This study confirms that under the sheltering conditions of artificial reefs, flow-field differences significantly affect the behavioral and physiological characteristics of fish. These findings provide an important scientific basis and theoretical reference for the ecological design and site selection of offshore wind farm pile foundations, as well as for assessing the impacts of habitat alterations following pile foundation construction on fishery resources.

## Simple Summary

Habitat restoration represented by artificial reefs is a vital means of protecting marine fishery resources. Offshore wind turbine foundations resemble artificial reefs in flow disturbance and shelter function, but the combined influence of the flow field and reef shelters on juvenile reef fish is still unclear. This study used juvenile crimson snapper (*Lutjanus erythropterus*) as the study species, set up four flow-velocity gradients with cubic artificial reefs as shelters, and analyzed their behavioral distribution and hepatic physiological responses to provide a theoretical reference for wind farm ecological construction and fishery habitat restoration. Flow velocity showed a positive correlation with fish oxygen consumption. Moderate 3 BL/s flow made juveniles stay stationary behind the reef most often and gather in the low-flow wake area. High 5 BL/s flow impaired reef sheltering, caused backward drift, and sharply increased lactic acid and cortisol. Low 1 BL/s flow induced the highest hepatic glucose, helping them resist mild flow stress. Artificial reef shelters can ease flow-induced physiological stress for juvenile snappers under suitable flow conditions, yet high flow will disrupt their normal behavior and metabolism.

## 1. Introduction

As a typical warm-water reef fish inhabiting the Indo-West Pacific, *Lutjanus erythropterus* is widely found in the South China Sea and the southern East China Sea. This species plays a dual role as both a vital economic resource for coastal fisheries and a key functional component of the rocky reef food web [1,2,3]. This species prefers complex habitats such as rocky and coral reefs. Reef structures and favorable local flow conditions can alleviate hydrodynamic stress and reduce locomotor energy consumption, thereby improving foraging efficiency and energy accumulation. Meanwhile, stable reef microhabitats protect individuals from predators and mitigate the disturbance of water flow, providing secure conditions for the spawning, development, and larval survival of *L. erythropterus*. Accordingly, the survival, growth, and reproduction of this species are highly dependent on the synergistic matching of local flow characteristics and habitat structure [4].

In recent years, the rapid development of offshore wind power industry has led to the widespread construction of underwater pile foundations in China’s coastal waters, which profoundly reshape the original flow field and habitat environment of local sea areas [5]. The flow-blocking and disturbing effects of pile foundations can form velocity-gradient zones and trailing vortex buffer zones, exhibiting highly similar flow-regulating and sheltering functions to artificial reefs, thereby creating novel artificial habitats [6]. The ecological impacts of such artificial structural modifications on reef-associated fish remain uncertain. On the one hand, these structures can optimize local habitat conditions and improve habitat complexity; on the other hand, abnormal flow disturbances may induce metabolic disorders and physiological stress in fish, threatening the survival of juvenile fish and the stability of fishery resources [7]. Clarifying the intrinsic relationships among offshore artificial structures, flow-field variations, and adaptive responses of reef fish is essential for the ecological impact assessment of offshore wind projects and the conservation of fishery resources.

Flow velocity is a critical environmental factor regulating fish behavioral decision-making, energy metabolism, and environmental adaptability [8]. In natural reef habitats, flow-refuge zones formed by complex reef structures effectively reduce swimming energy consumption and metabolic costs, serving as key supports for fish to adapt to hydrodynamic fluctuations [9,10]. Previous studies have demonstrated that flow velocity is significantly correlated with fish growth, feeding performance, and energy utilization efficiency. Abnormal flow fluctuations force fish to sustain continuous swimming against currents, resulting in behavioral disorder, energy consumption, and physiological stress, which substantially impair fish health and survival [11,12]. When exposed to hydrodynamic stress, fish initially achieve adaptive regulation through behavioral adjustments, including spatial distribution and locomotor rhythm modification, followed by sequential changes in oxygen consumption rate, energy substrates, and stress hormones. The synergistic behavioral and physiological modulation constitutes the core adaptive mechanism of fish in response to flow field variations [13,14]. Among the key indicators, oxygen consumption rate intuitively reflects metabolic intensity; glucose and lactate characterize aerobic and anaerobic metabolic levels, respectively; and cortisol serves as a precise biomarker for quantifying environmental stress intensity. Combined, these indicators effectively evaluate fish flow-stress responses and environmental adaptability [15,16,17].

Most existing studies on fish responses to flow velocity focus on simple open-flow environments and primarily explore the independent effects of single flow gradients on fish physiology and growth, while largely neglecting the widespread ecological process of shelter-flow coupling in natural and engineering scenarios [18,19]. There is a lack of systematic research on the synergistic behavioral and physiological response mechanisms of fish under the combined effect of artificial shelters and variable flow fields. Accordingly, this study selects juvenile *L. erythropterus*, a typical reef-dependent fish species, as the research object. Artificial reefs are adopted to simulate the sheltering and habitat-disturbing functions of offshore wind pile foundations. Centering on the core scientific issue regarding how flow-field variations regulate fish behavioral and physiological performances in compound artificial habitats, this study reveals the behavioral and physiological characteristics of juvenile *L. erythropterus* in response to flow changes within sheltered habitats. The findings provide a theoretical basis for ecological site selection, structural optimization of offshore wind farm pile foundations, and the conservation of coastal fishery resources.

## 2. Materials and Methods

### 2.1. Experimental Fish and Breeding Conditions

*L. erythropterus* used in this experiment were from a single batch and purchased from Shenzhen Haiyuan Biotechnology Co., Ltd., Dapeng New District, Shenzhen, Guangdong, China. The experimental fish had a body length of 9.93 ± 1.48 cm, body height of 4.23 ± 0.21 cm, and body weight of 14.93 ± 1.78 g. A total of 500 juveniles of *L*. *erythropterus* were acclimated in an industrialized flow-through aquaculture facility. In this once-through system, ambient seawater continuously flowed into the rearing tanks, and overflow wastewater was directly discharged without filtration or water recirculation. They were fed commercial pelleted compound feed for snapper to satiation twice daily (at 8:30 and 17:30), and 40% of the water in the rearing tanks was exchanged every two days. The water temperature was maintained at 28 ± 1 °C, salinity at 30.0‰, and pH at 7.98 ± 0.10. Natural light was used as the light regime. Dissolved oxygen (DO) was kept at ≥7 mg/L. Continuous aeration was provided in the acclimation tanks for 24 h per day. The experiment was initiated after a 7-day acclimation period. Fish were fasted for 24 h prior to the start of the experiment. The experiment was initiated on 23 January 2025.

### 2.2. Experimental Setup and Flow Rate Control

A circulating water flume (Model FBT-10X, Qingdao Haixing Instrument Co., Ltd., Qingdao, China) designed for assessing fish swimming performance and related physiological metrics was employed in this experiment. The test section of the flume measured 50 cm × 50 cm × 120 cm, with a testable flow velocity range of 5–120 cm/s. The system is capable of operating in timed modes (1–999 min) and constant-velocity intermittent modes (10–120 cm/s). To prevent water splashing during the experiment, the water level was maintained 10 cm below the upper edge of the flume. Water flow was driven by an underwater propeller, and a honeycomb flow straightener was installed to generate a stable current within the test section. The flow velocity was regulated by adjusting the power output of the machine. Additionally, Hikvision network cameras (Model DS-2CD3T47EWDV3-L, Hangzhou Hikvision Digital Technology Co., Ltd., Hangzhou, China) were mounted at the front and top of the flume to observe and record the behavior of juvenile *L. erythropterus*.

The flow velocity of the experimental setup was measured. For this circulating water flume, the flow velocity in the test section can be regulated by adjusting the motor power. To facilitate precise velocity control during the experiment, the relationship between the actual flow velocity and the motor power was determined to establish a calibration curve. Furthermore, based on the actual flow velocity range of the flume, it was confirmed that the system basically meets the testing requirements for juvenile *L. erythropterus*.

This experiment analyzed the relationship between flow velocity in the experimental area and regulator power. The fitted relationship is as follows:

Y = 1.801 × X + 2.088 (R^2^ = 0.9852, *p* < 0.0001)

where Y is the flow velocity in the experimental area (cm/s), and X is the regulator power (W).

**Figure 1:**
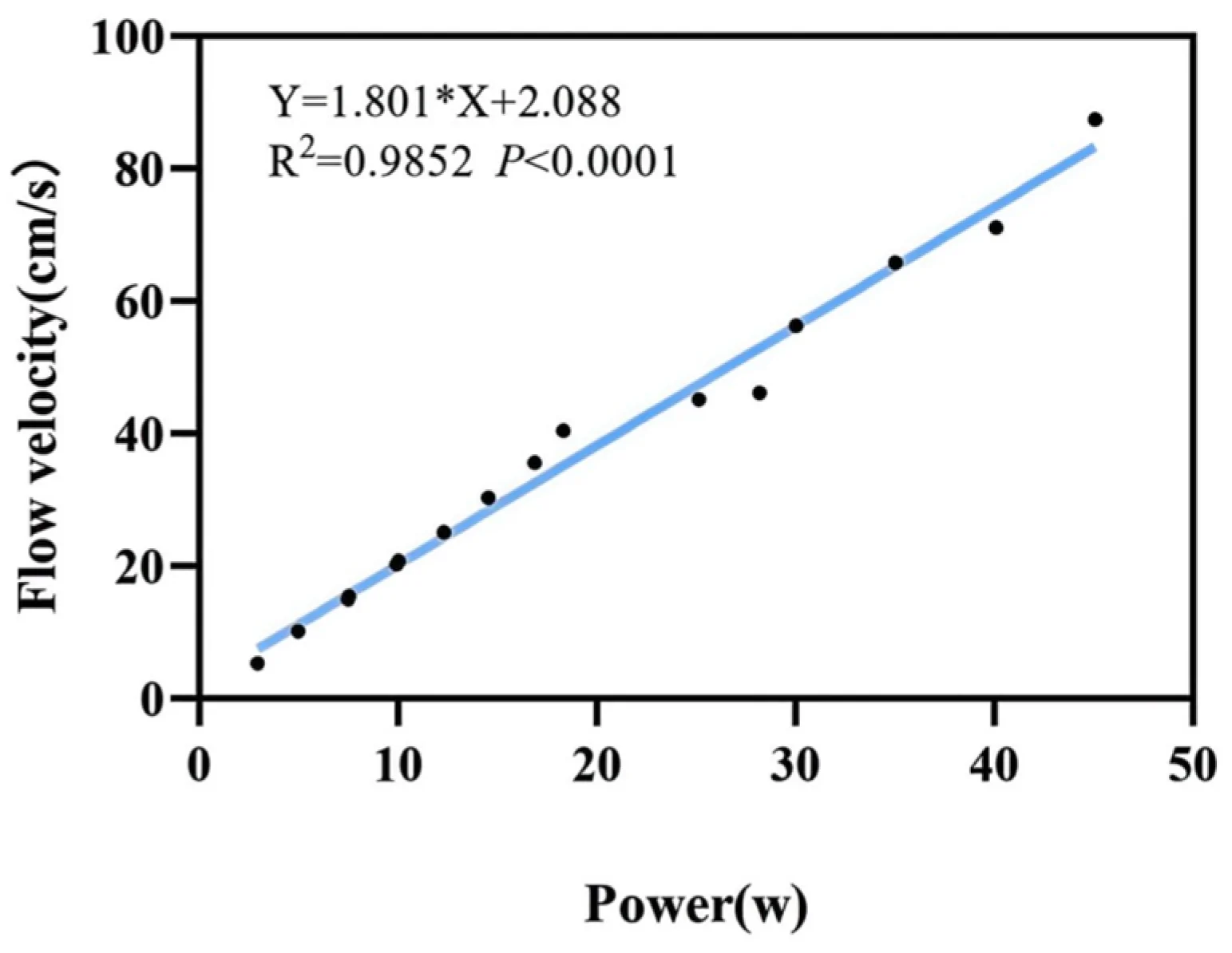
Power–flow rate relationship.

The flow velocity in the experimental area showed a linear positive correlation with regulator power (R^2^ = 0.9852, Figure 1). As the regulator power increased, the flow velocity in the experimental area increased accordingly.

### 2.3. Determination of Critical Swimming Speed and Oxygen Consumption Rate

To determine the gradient values of flow velocity for the experiment, the present study conducted stepped velocity tests to measure the absolute critical swimming speed (*U_acrit_*) and relative critical swimming speed (*U_rcrit_*) of juvenile fish, with the relative critical swimming speed adopted as the upper limit of the experimental flow velocity. For the determination of *U_acrit_*, a single fish was placed in the test section and exposed to an initial flow velocity of 0.5 BL/s for 30 min. Subsequently, the velocity was increased by 0.5 BL/s every 30 min until exhaustion [18]. Fatigue was defined as the fish being swept against the downstream honeycomb screen and unable to resume swimming for 20 s. The final swimming velocity and elapsed time were recorded at this point. Each fish was used only once and not reused to prevent prior fatigue from affecting the experimental results.

*U_acrit_* was calculated based on the following formula [19]:

*U_acrit_* = *U* + ∆*U* × *t*/∆*t*

where *U* is the highest velocity (cm/s) maintained for a full time interval; ∆*U* is the velocity increment (cm/s); *t* is the swimming time (min) at the final velocity; and ∆*t* is the prescribed time interval (min).

The *U_rcrit_* was expressed as the absolute critical swimming speed divided by body length, and calculated using the following formula [19]:

*U_rcrit_* = *U_acrit_*
/(*BL*)

The oxygen consumption rate (mg O_2_·h^−1^·kg^−1^) was measured in a circulating water flume with a water volume of 0.24 m^3^. Twenty juvenile *L. erythropterus* were introduced per experimental unit. Before testing, the dissolved oxygen (DO) of the water was adjusted to above 7.00 mg/L. The system was sealed after fish acclimation to reduce oxygen exchange at the water–air interface. The water temperature was maintained at 28 ± 1 °C during the 2 h trial. DO was continuously monitored with an oxygen meter and kept above 2.0 mg/L throughout the experiment. The initial and final DO values were recorded to calculate total oxygen consumption for the 20 fish. The number of fish was identical across all velocity treatments. The 20 juveniles in each unit represented a single experimental unit, and the obtained oxygen consumption rate denoted group-level oxygen consumption. Three independent experimental replicates were performed for each flow velocity treatment. The oxygen consumption rate was calculated using the following formula [20]:

*Oxygen consumption rate* = *V(d(DO)/t)*/*M*

where *V* is the volume of the swimming flume (L); *M* is the mass of the selected *L. erythropterus* juveniles; and *d(DO)*/*dt* is the slope of the linear regression of *DO* over time. The background oxygen consumption rate of the system (measured without fish) was less than 1% of the *L. erythropterus* oxygen consumption rate; therefore, its effect was considered negligible.

### 2.4. Shelter Structure and Behavioral Experiments

**Figure 2:**
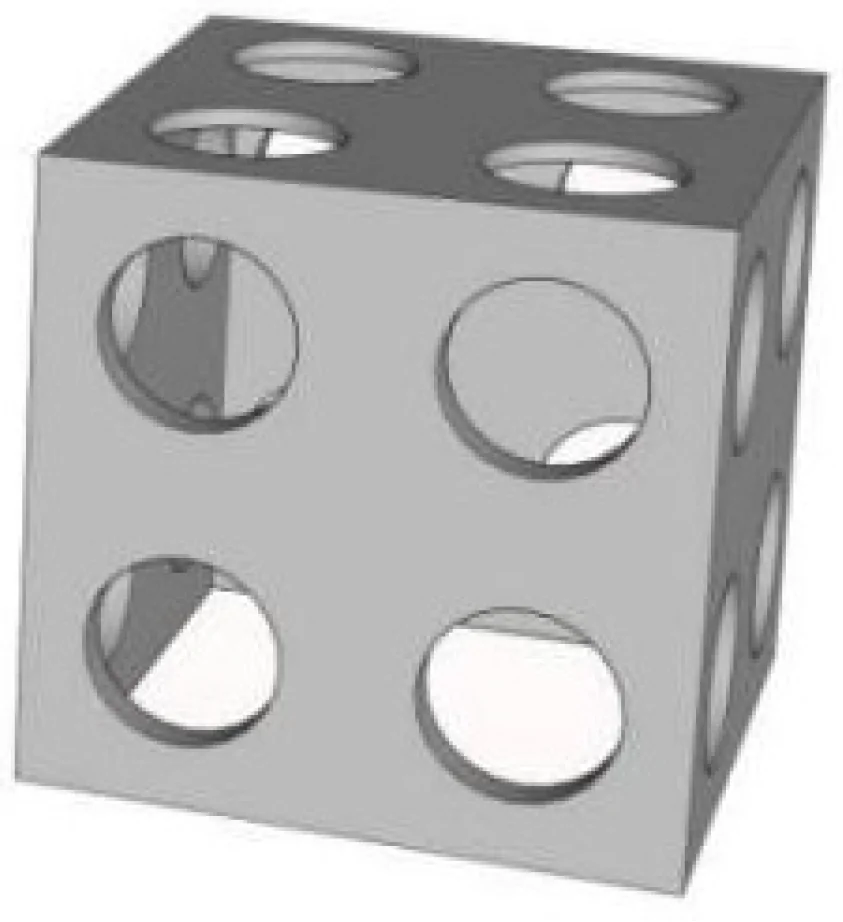
Schematic diagram of the artificial reef model.

To simulate the habitat alteration and shelter conditions caused by artificial underwater structures, this experiment used a cubic artificial reef measuring 15 cm × 15 cm × 15 cm (length × width × height) as the shelter structure (Figure 2). This reef configuration, with optimized opening shape and opening height for *L*. *erythropterus*, was determined based on our team’s previous research [21]. Prior to the experiment, the reef was placed vertically in the test section, 30 cm downstream from the upstream honeycomb screen, to create a localized shelter habitat. During the experiment, ten naïve juveniles were randomly introduced into each flow velocity group. Upon completion of the experiment, these fish were transferred to designated rearing tanks for communal rearing, and no further experiments were performed on them thereafter. Their behavior was monitored in real-time using front- and top-mounted network cameras (DS-2CD3T47EWDV3-L, Hangzhou Hikvision Digital Technology Co., Ltd., Hangzhou, China), and their activities were continuously recorded for 120 min using a high-speed camera (GoPro HERO 11 Black, GoPro, Inc., San Mateo, CA, USA). High-speed videos of *L. erythropterus* swimming in the presence of the cubic reef were collected.

Based on preliminary experiments and the tolerance range of the juveniles, four flow velocity treatments were designed: 0 BL/s (0 cm/s), 1 BL/s (9.93 cm/s), 3 BL/s (29.79 cm/s), and 5 BL/s (49.65 cm/s). The 0 BL/s (0 cm/s) treatment served as the static control. Prior to each experiment, *L. erythropterus* were placed in the flume and acclimated at a low velocity of 1 BL/s for 30 min. The locomotor behavior of the fish was then evaluated under the four flow velocities (0, 1, 3, and 5 BL/s).

### 2.5. Behavioral Indicators

Video capture was performed using LoliTrack5 Webcam Software (Version 5, Loligo^®^ Systems, Viborg, Denmark). All video recordings were analyzed, and fish locomotor behaviors were determined by a single observer (Yu Guo), ensuring consistent behavioral definition criteria for juvenile *L. erythropterus* throughout the entire analysis process. Based on video recognition, the swimming behavior of fish moving behind a cubic reef is categorized into three states: stationary (maintaining position for more than 5 s), forward movement (upstream displacement exceeding 10 cm), and backward movement (downstream displacement exceeding 10 cm) [22]. Each trial randomly selected 5 individuals for behavioral video analysis, with more than 10 video segments covering three types of locomotor states recorded for each selected fish. The experiment was repeated three times using biologically independent batches of juveniles. Fish within the same flume tank were treated as one experimental unit; individual fish in the same tank were not regarded as statistically independent observations. For statistical analysis, behavioral metrics from the five fish in a single trial were averaged to generate one replicate datum. The time percentage of each locomotor state was used to compare differences among the 0, 1, 3, and 5 BL/s groups. The calculation method for the time percentage of each state is as follows:

The time percentage of each locomotor state (forward movement and backward movement) at 0 BL/s (1 BL/s, 3 BL/s, and 5 BL/s) = (Time observed for stationary behavior behind the cubic reef at 0 BL/s)/(Total time observed for all behaviors at 0 BL/s).

**Figure 3:**
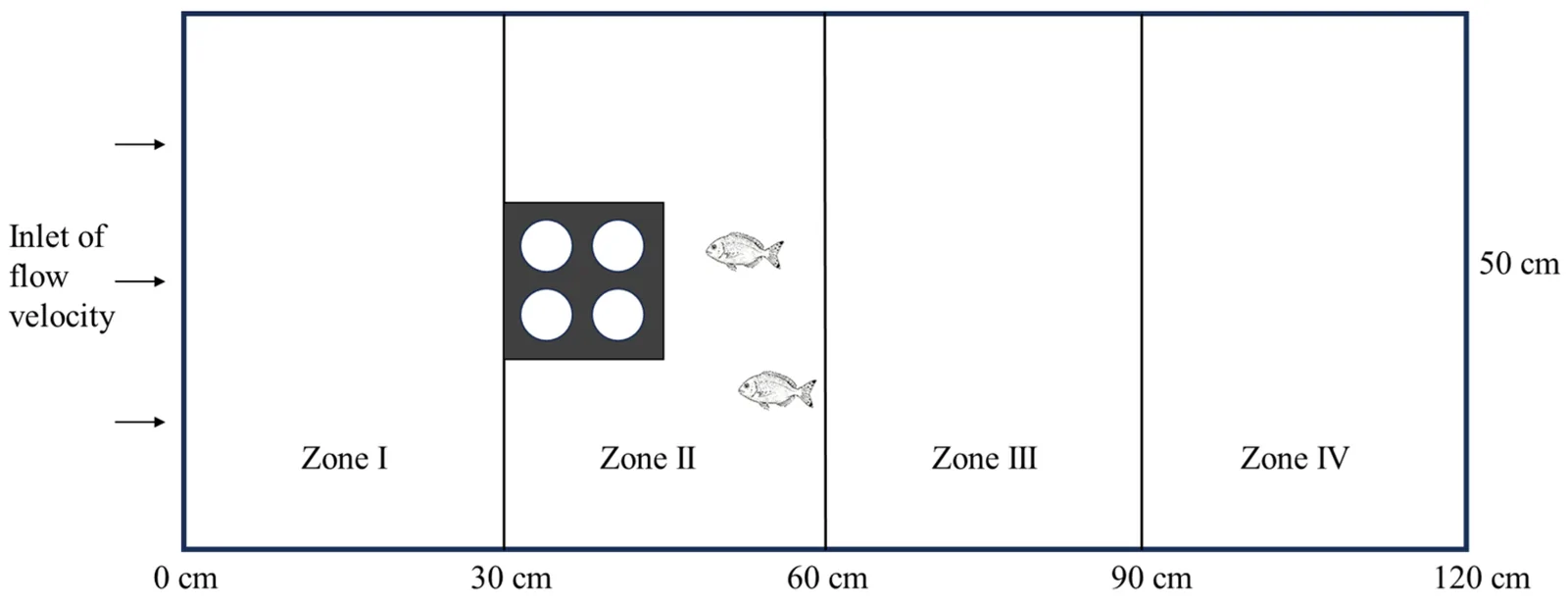
Swimming ability measurement circulating tank division diagram.

Additionally, based on the video data, screenshots were captured every 30 min to record the spatial positions of *L. erythropterus* across different zones. The flume was divided longitudinally along the flow direction into four zones: upstream zone (Zone I, 0–30 cm), reef zone (Zone II, 30–60 cm), near-downstream zone (Zone III, 60–90 cm), and far-downstream zone (Zone IV, 90–120 cm), as illustrated in Figure 3. The preferred zones of *L. erythropterus* under different flow velocities were evaluated by calculating the mean distribution rate (MDR) in each zone. The MDR is calculated as follows:

$$\mathrm{MDR}=\frac{1}{\mathrm{mn}}\sum_{i=1}^{m}n_{i}\times 100\%$$

where $n_{i}$ represents the number of individuals of the target fish species in a specific zone during the *i*-th observation; *n* is the total number of observations; and *m* denotes the total number of individuals in the experimental group [23].

### 2.6. Sample Collection and Physiological Index Determination

The flow velocity grouping design was identical to that of the behavioral experiments. To minimize the effects of environmental stress and individual variations, 10 naïve juveniles were placed in the swimming performance flume for a 1 h acclimation period. The flow controller was then activated to set the target flow velocity, and the fish swam continuously at this velocity for 1 h. At the end of each trial, seven juveniles were randomly sampled from the flume for physiological measurement. Three biologically independent replicates were performed, each replicate using a separate batch of juvenile fish. All fish within a single flume were treated as one experimental unit, and individual fish within the same tank were not regarded as statistically independent observations. *L. erythropterus* were then rapidly anesthetized using an MS-222 solution (150 mg/L). Liver tissues were harvested, transferred into sterile cryogenic vials, immediately snap-frozen in liquid nitrogen, and subsequently stored at −80 °C until further analysis.

Prior to detection, sample pretreatment was performed. Briefly, liver tissues were accurately weighed and homogenized in pre-cooled normal saline at a fixed ratio. The homogenate was centrifuged at low temperature and high speed, and the supernatant was collected for the determination of hepatic glucose (GLU), lactic acid (LD), and cortisol (COR) [24]. All detection kits were purchased from Nanjing Jiancheng Bioengineering Institute (Nanjing, China). GLU was determined by the glucose oxidase method, LD by the colorimetric method, and COR by the double-antibody sandwich ELISA method, with all operations strictly conducted in accordance with the kit instructions. Each sample was measured in three technical replicates, and the average value was adopted as the final result.

Optical density (OD) values were measured using a SpectraMax Plus 384 microplate reader (Molecular Devices, San Jose, CA, USA) at wavelengths of 505 nm for GLU, 530 nm for LD, and 450 nm for COR, respectively. Standard curves were established using gradient standard solutions supplied with the kits, with a coefficient of determination R^2^ > 0.990 to guarantee quantitative accuracy. All measured values exceeded the minimum detection limits of the kits (GLU: 0.01 mmol/gprot; LD: 0.02 mmol/gprot; COR: 0.1 ng/mgprot). The intra-assay coefficient of variation was controlled below 5%, and the inter-assay coefficient of variation was below 10%, meeting standard biochemical detection requirements. The total protein content of liver tissue was determined via the Coomassie brilliant blue method to correct the detection results of all physiological indicators. The final results were expressed in units of mmol/gprot (GLU, LD) and ng/mgprot (COR).

### 2.7. Statistical Analysis

Data sorting and preliminary processing were performed using Excel 2016, and statistical analysis was conducted via SPSS 26.0. A one-way analysis of variance (ANOVA) was adopted as the statistical model for all measured indices in this study, with flow velocity set as the single independent variable and behavioral and physiological parameters as dependent variables. The null hypothesis assumed no significant differences in the tested indices among different flow velocity treatments, while the alternative hypothesis assumed the presence of significant intergroup differences. All experimental treatments were based on completely independent biological replicates without repeated measurement or pseudoreplication, and behavioral and physiological datasets were analyzed independently to avoid statistical confounding. Intergroup differences were tested at a significance level of *p* < 0.05. Once a significant main effect was detected, Duncan’s multiple range test was applied for post hoc pairwise comparison. All experimental results were presented as mean ± standard deviation (mean ± SD).

## 3. Results

### 3.1. Extreme Flow Rate and Oxygen Consumption Rate

The *U_acrit_* of *L. erythropterus* was measured to be 53.04 ± 5.27 cm/s, with a relative *U_crit_* of 5.34 ± 0.53 BL/s. Accordingly, the highest flow-velocity treatment in this experiment was set at 5 BL/s.

**Figure 4:**
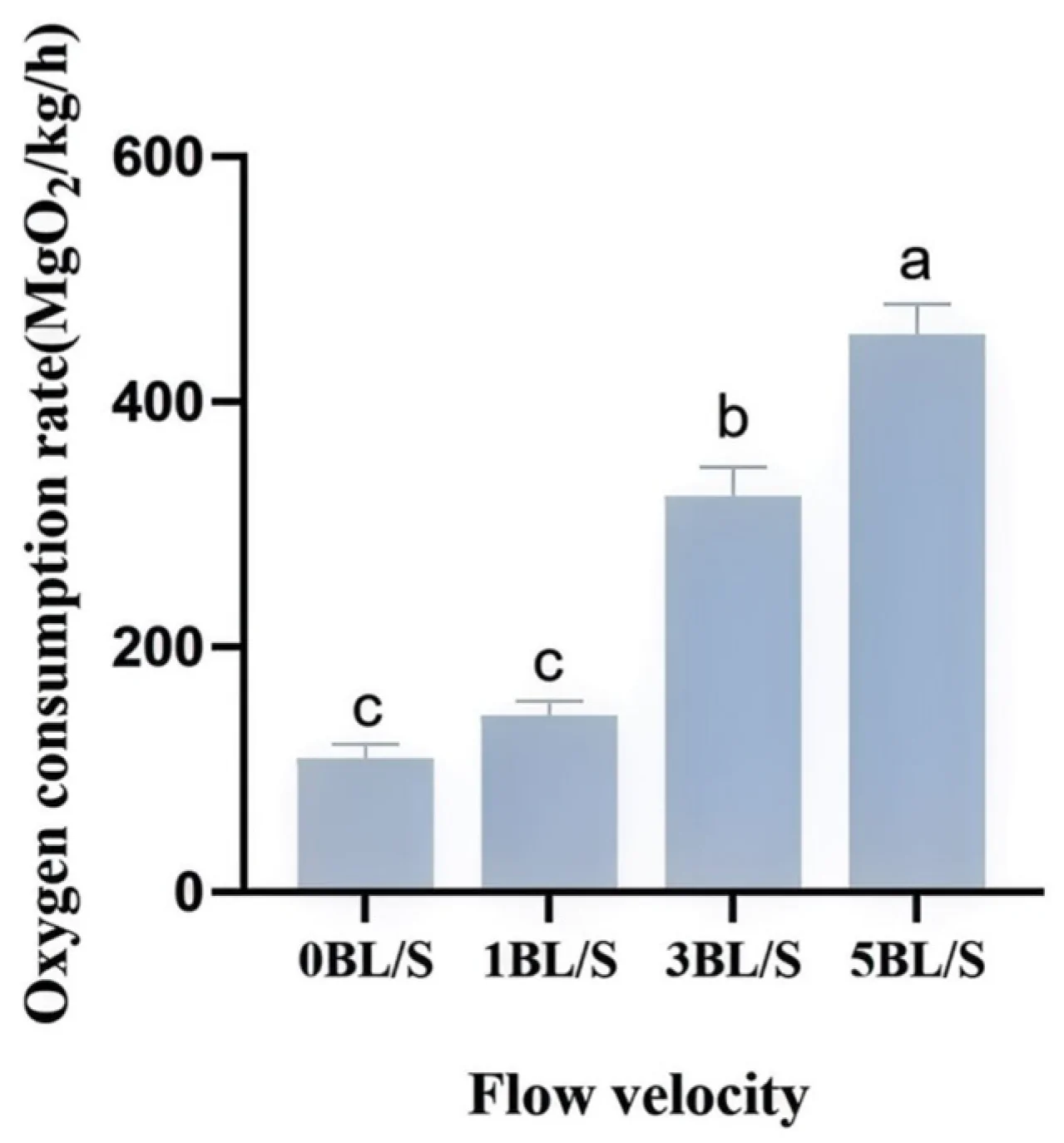
Oxygen consumption rate of *L. erythropterus* at different flow rates. Values are presented as mean ± standard error of the mean (SEM). Different lowercase letters above bars indicate significant differences among groups (*p* < 0.05), whereas the same lowercase letters indicate no significant difference (*p* > 0.05).

All flow velocity treatments in this experiment were set with identical biological replicates, with a valid sample size of n = 3 for each group. The error bars in the figures represent the standard error of the mean (SEM). The oxygen consumption rate of *L. erythropterus* at varying flow velocities is presented in Figure 4. The oxygen consumption rate increased with the rise in flow velocity, and significant differences were observed among the different treatment groups (*p* < 0.05). Specifically, rates at 0, 1, 3, and 5 BL/s were 109.25 ± 10.87, 143.59 ± 11.87, 323.39 ± 22.68, and 455.20 ± 24.71 mg O_2_·h^−1^·kg^−1^, respectively, reaching the highest level at 5 BL/s. This indicates that as flow velocity increases, the swimming intensity of *L. erythropterus* is significantly enhanced, accompanied by a continuous increase in aerobic metabolic activity.

### 3.2. The Influence of Flow Velocity on the Movement State of L. erythropterus

**Figure 5:**
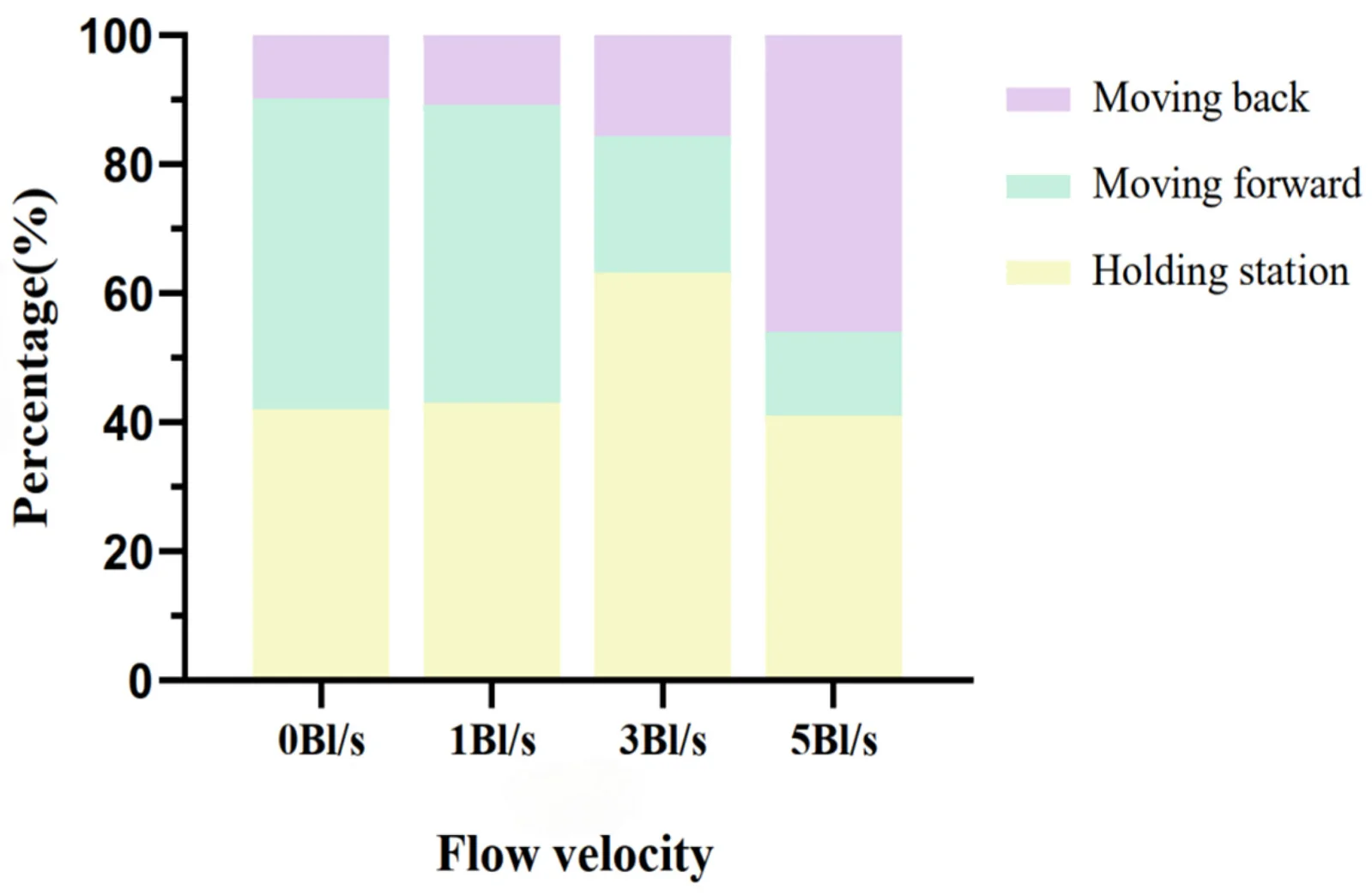
Percentage of *L. erythropterus* movement at different flow velocities.

The percentages of time holding station, moving forward, and moving back by *L. erythropterus* behind a cubic reef were related to flow velocity, demonstrating a significant flow-velocity dependence in their behavioral states around the reef (Figure 5). The behavioral patterns at 0 BL/s and 1 BL/s were similar, with the fish remaining stationary or moving forward for most of the time. When the flow velocity increased to 3 BL/s, the proportion of time juveniles spent resting peaked at 63.23%, which was significantly higher than that of the 1 BL/s (43.02%) and 5 BL/s (41.09%) groups.

When the flow velocity was further increased to 5 BL/s, the proportion of time spent moving backward reached its peak, approaching the level of resting time. Additionally, experimental observations revealed that some juveniles exhibited behaviors such as pressing against the netting and struggling to maintain controlled swimming. This indicates that at high flow velocities, the sheltering effect of the reef is no longer sufficient to counteract hydrodynamic forces. The juveniles are unable to stably remain within the sheltered zone and are forced downstream by the current. Concurrently, they must expend considerable energy in an attempt to hold their position, leading to disordered locomotor patterns. These behavioral disruptions are consistent with the aforementioned findings that MO_2_ gradually increases with rising flow velocity, reflecting higher energy expenditure.

### 3.3. Spatial Distribution of L. erythropterus at Different Flow Velocities

**Figure 6:**
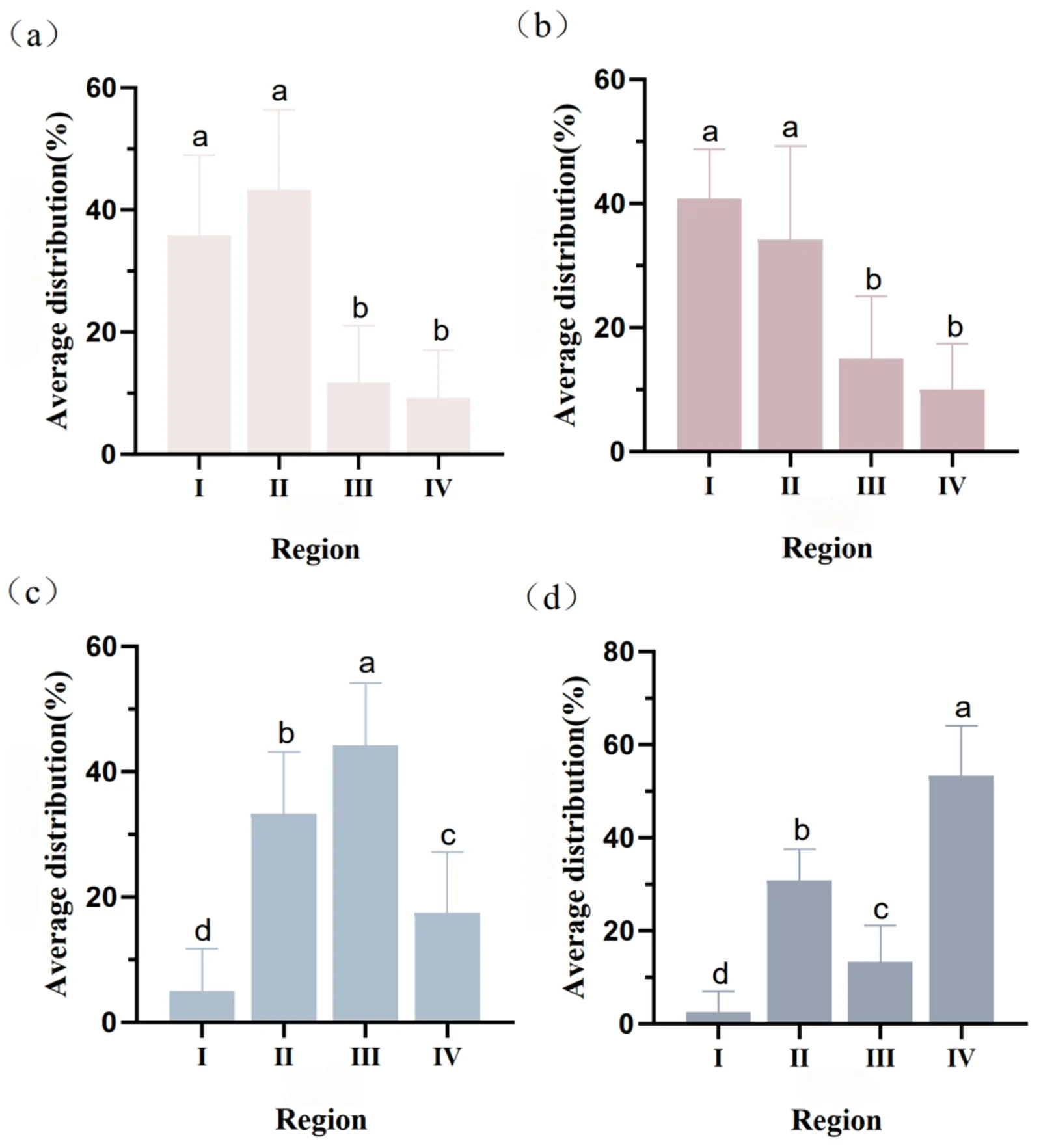
Mean distribution rate in each region at different flow rates: (**a**) 0 BL/s; (**b**) 1 BL/s; (**c**) 3 BL/s; (**d**) 5 BL/s. Values are presented as mean ± standard error of the mean (SEM). Different lowercase letters above bars denote significant differences among zones (*p* < 0.05), while identical lowercase letters denote no significant difference (*p* > 0.05).

The MDR results visually demonstrate how the utilization strategy of juveniles for shelter habitats shifts in response to changing flow fields. As shown in Figure 6, at flow velocities of 0 and 1 BL/s, the fish were primarily distributed over a broad area upstream of the cubic reef (Zone I) and within the reef zone itself (Zone II). This indicates that under zero or low flow conditions, juveniles tend to remain active around the reef, utilizing it as a core area for exploration and resting, without exhibiting strong flow-avoidance aggregation.

At a flow velocity of 3 BL/s, the fish were primarily distributed in Zone III downstream of the cubic reef. This result clearly demonstrates that under moderate hydrodynamic stress, juveniles actively selected the low-flow recirculation zone behind the reef as a sheltered space. By adjusting their spatial distribution to avoid the high-velocity mainstream area, they exhibited an active behavioral adaptation strategy.

At the high flow velocity of 5 BL/s, the distribution of juveniles shifted further downstream, concentrating primarily in the far-downstream Zone IV. This was not an active choice for sheltering, but rather a consequence of the high mainstream velocity and the limited shielding range of the reef. Unable to remain stably in the leeward zone, the juveniles were swept by the strong currents to areas further downstream. This observation is entirely consistent with the increased proportion of backward swimming time in their locomotor patterns, reflecting the passive displacement caused by the failure of sheltering effects under high flow velocities.

### 3.4. The Effect of Flow Velocity on Physiological Indicators of L. erythropterus

Changes in physiological indicators further reveal the physiological regulation and stress status of juveniles under different flow fields.

**Figure 7:**
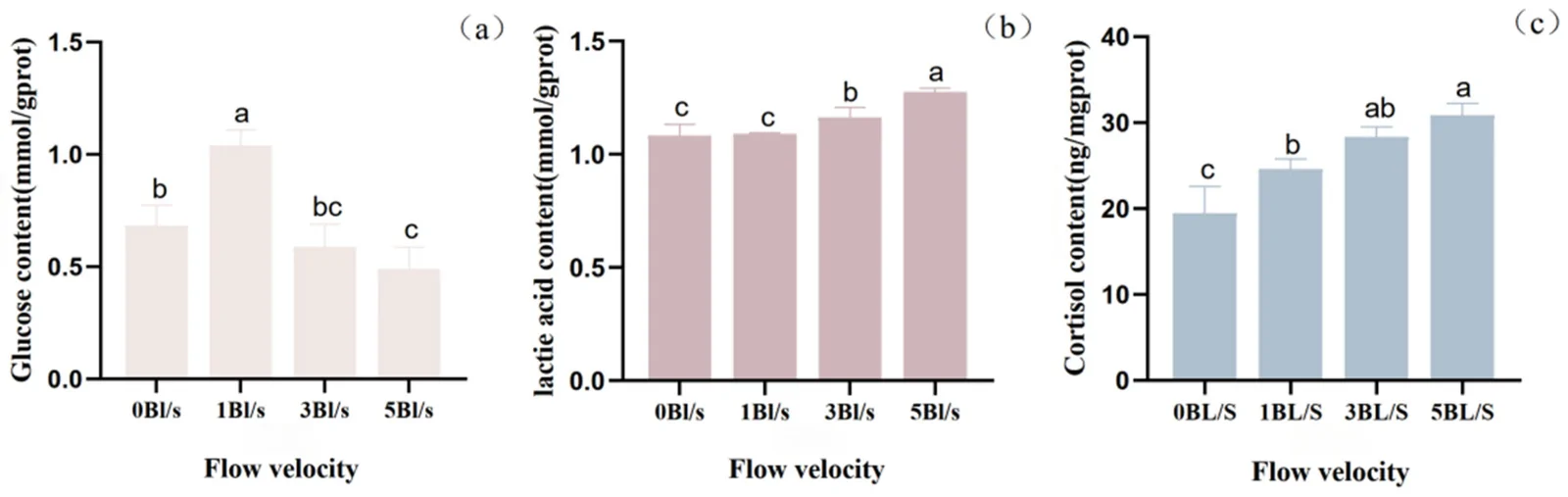
Physiological indicators: (**a**) GLU; (**b**) LD; (**c**) COR. Values are presented as mean ± standard error of the mean (SEM). Different lowercase letters above bars indicate significant differences among flow-velocity groups (*p* < 0.05), and the same lowercase letters indicate no significant difference (*p* > 0.05).

The GLU contents of the experimental groups under different flow velocities are presented in Figure 7a. The GLU content in the 1 BL/s group was significantly higher than that in the other groups (*p* < 0.05), whereas it was relatively low in the 5 BL/s group. This indicates that under low-flow stress, the organism elevates blood GLU levels by breaking down glycogen to reserve energy for potential locomotor demands; however, when the flow velocity becomes excessively high, GLU is rapidly consumed to sustain high-intensity swimming, resulting in a decrease in its content.

Hepatic LD content in juvenile *L. erythropterus* exhibited a continuous upward trend with increasing flow velocity, as shown in Figure 7b. The LD contents in the 0 BL/s and 1 BL/s groups were relatively low, with no significant difference between them (*p* > 0.05). Conversely, the highest LD content was observed in the 5 BL/s group. Under this flow condition, some experimental fish displayed behaviors such as pressing against the netting and struggling to maintain swimming control, indicating enhanced metabolism and the onset of exercise-induced fatigue.

As a core stress hormone, COR levels showed significant differences among the various flow velocity groups (*p* < 0.05, Figure 7c). Hepatic COR content in juvenile *L. erythropterus* increased progressively with flow velocity, reaching its peak at 5 BL/s. These findings confirm that as hydrodynamic stress intensifies—particularly when the sheltering effect becomes ineffective—the juveniles mount an enhanced physiological stress response.

## 4. Discussion

### 4.1. The Influence of Flow Velocity on the Behavioral Characteristics of L. erythropterus

Swimming performance is a critical determinant of fish survival in aquatic environments. Assessments of swimming capacity typically involve metrics such as critical swimming speed and relative critical swimming speed. As widely used measurements, these parameters play a vital role in evaluating sustained swimming ability, thereby providing crucial insights into the ecology and physiology of fish [25]. Furthermore, *U_acrit_* and *U_crit_* play a crucial role in fish locating potential mates, foraging, and navigating to suitable habitats [26]. Swimming capacity and energy expenditure vary among different migratory fish species. In this study, the *U_acrit_* of juvenile *L. erythropterus* was 53.04 ± 5.27 cm/s, with a *U_crit_* of 5.34 ± 0.53 BL/s. These values are notably higher than those observed in several other species, including *Pomacentrus amboinensis*, *Pomacentrus mollucensis*, *Ostorhinchus compressus*, *Sphaeramia nematoptera*, *Amphiprion percula*, and *Amphiprion melanopus* [25]. This indicates that *L. erythropterus* possesses a moderate level of critical swimming capacity. The significant variations in swimming performance observed among different fish species may be attributed to their specific habitats, morphological structures, developmental stages, and energy metabolism characteristics. Although critical swimming speed and relative critical swimming speed may provide less information regarding swimming physiology and field swimming speeds compared to other velocity definitions, they both serve as reliable estimates of overall swimming capacity because these two metrics encompass both aerobic and anaerobic swimming. Swimming performance is a vital life-history trait for fish survival, yet it is not the sole determinant of their in situ survival. Multiple factors, including food availability, accessibility of refuge habitats, environmental stress, interspecific competition, and predation pressure, jointly regulate fish survival performance [27,28]. Therefore, when analyzing fish adaptability based on critical swimming speed, we should not infer field survival outcomes solely from swimming-performance metrics; comprehensive discussions incorporating these multiple survival-related factors are also required.

Reef-dwelling fish inhabiting environments with specific types and sizes of obstacles may utilize these structures to enhance their swimming capacity, thereby reducing energy expenditure [29]. In this study, flow velocity exerted varying effects on fish locomotion. At low flow velocities, fish exhibited a transition between stationary and forward swimming states, with their movement patterns resembling those of free-swimming behavior. Compared to 1 BL/s, at 3 BL/s the school was confined to a smaller area; this spatial restriction may serve as an adaptive strategy to avoid excessive energy expenditure associated with exploratory or turning behaviors [22]. However, as flow velocity increased to 5 BL/s, the percentage of time spent retreating increased. Fish posture became increasingly unstable and complex. Juvenile *L. erythropterus* tended to remain in the downstream area of the cubic reef, likely because intensified turbulence made it more difficult to maintain a stationary position. The hydrodynamic characteristics induced by the artificial reef and the body size of the fish are critical determinants of their swimming behavior [30]. However, this parameter was not measured in the present study. Further research is warranted to investigate the relationships among fish size, swimming behavior, and the hydrodynamic properties of the flow around obstacles.

Swimming status and locomotor patterns of the fish can serve as indirect indicators of their adaptability [31]. This experiment revealed that juvenile *L. erythropterus* exhibit intermittent swimming at low flow velocities, primarily relying on pectoral fin beats and pelvic fins to maintain balance. Previous studies have demonstrated that this intermittent locomotion helps reduce energy expenditure [22]. In this study, juvenile *L. erythropterus* exhibited the highest frequency of discontinuous swimming behavior at 0 BL/s and 1 BL/s, indicating greater adaptability to these flow velocities.

### 4.2. The Effect of Flow Velocity on Physiological Metabolism and Stress Level of L. erythropterus

Fish performance during a series of swimming activities is closely associated with respiration and oxygen utilization. Consequently, the oxygen consumption rate has become an integral component of physiology, bioenergetics, and ecology. Generally, oxygen consumption rate is positively correlated with the metabolic activity of an organism [14,32]. Changes in flow velocity induce distinct locomotor patterns in fish, thereby leading to oxygen consumption [33]. The measurement of oxygen consumption rate holds significant research implications for understanding physiological changes in fish under varying flow velocities, as well as their metabolic shifts across different developmental stages. Generally, the oxygen consumption rate of fish swimming in still water is significantly lower than that when swimming in flowing water [34]. This indicates that flow-induced changes in swimming significantly increase the energy expenditure of the organism. Consistent with this, our experiment measured the oxygen consumption rate of juvenile *L. erythropterus* within the testing section of a swimming respirometer and obtained similar results. Furthermore, tail-beat frequency can serve as an indirect indicator of a fish’s adaptability to water flow. Most caudal-fin-propelled fish increase their swimming speed by elevating their tail-beat frequency [8]. However, tail-beat frequency was not measured in this study. Future research should integrate tail-beat frequency with swimming speed and physiological changes for further analysis.

GLU is a primary indicator for assessing energy expenditure during fish locomotion. When subjected to stress, fish experience a significant increase in energy demands, triggering a series of complex neuroendocrine and tissue-level physiological changes. Under these conditions, GLU serves as the main functional substrate. Generally, blood GLU levels are maintained in dynamic homeostasis, and moderate exercise helps regulate these levels [35]. In this experiment, the GLU content of juvenile *L. erythropterus* at a flow velocity of 1 BL/s was higher than that in the 0 BL/s group. This may be because when fish perceive increased water flow and altered activity demands, their bodies initiate stress response mechanisms to cope with potentially elevated energy expenditure. To meet these heightened energy requirements, the fish mobilize internal energy reserves. As a key organ for energy storage and metabolism, the liver can break down glycogen into GLU and release it into the bloodstream, thereby causing an increase in hepatic GLU levels during this phase to satisfy the growing energy demands [36]. However, when the flow velocity exceeds 3 BL/s, GLU levels begin to decrease. During locomotion at higher flow velocities, increased energy demands lead to greater GLU consumption for aerobic respiration, resulting in a gradual decline in GLU content. Furthermore, endogenous GLU levels may also be influenced by multiple factors, including fish species, body size, sampling site, physiological state, and experimental timing.

Previous studies have shown that when fish experience higher swimming intensity, their energy production shifts gradually from aerobic respiration to anaerobic respiration. During this transition, lactic acid is generated via glycolysis. The accumulated lactic acid builds up in muscle tissue and blood, triggering fatigue and ultimately impairing swimming performance and whole-body metabolism. In this experiment, exposure to high flow velocities of 3 and 5 BL/s resulted in a significant increase in LD levels (*p* < 0.05), indicating that the fish were likely in a state of fatigue. Flodmark et al. found that in juvenile brown trout (*Salmo trutta*) subjected to flow fluctuations, blood COR and GLU levels increased as the magnitude of flow fluctuation intensified. Similarly [37], Milligan et al. reported a significant increase in LD levels in the liver and pancreas of rainbow trout (*Oncorhynchus mykiss*) following exhaustive exercise. These findings are consistent with the results of our present study.

COR is generally used as an indicator of exposure to stressors [15,38]. In the present study, high flow velocities induced a stress response in *L. erythropterus*, leading to elevated COR concentrations. Notably, when the flow velocity reached 5 BL/s, COR levels were significantly higher than those in the other treatment groups (*p* < 0.05). However, at 3 BL/s, while COR levels continued to rise, GLU content began to decrease. Previous research has demonstrated that COR levels are correlated with the duration of stressor exposure [39]. We hypothesize that this phenomenon may be attributed to prolonged stress, which causes excessive energy depletion and subsequently leads to a decline in GLU levels, even as COR continues to accumulate.

### 4.3. The Behavioral Physiological Synergistic Response Mechanism of Flow Velocity to L. erythropterus Under Habitat Protection

Flow is a critical and complex ecological factor in fish habitats. It stimulates sensory perception, thereby triggering specific locomotor patterns and response mechanisms. Furthermore, flow exerts multidimensional ecological effects, directly or indirectly regulating fish behavioral traits and physiological ecology [40]. For instance, Danio rerio are known to utilize specialized sensory and metabolic physiological mechanisms to modulate their responses in fast-flowing waters, ultimately reducing the energetic costs of survival [41].

The response of juvenile *L. erythropterus* to flow-velocity variations follows a synergistic regulatory mechanism, characterized by behavioral adaptation preceding physiological and metabolic support. An initial increase in flow velocity prompts juveniles to make external adjustments by altering their locomotor patterns and spatial positioning. When behavioral regulation can no longer offset the hydrodynamic pressure, the organism initiates energy metabolism reprogramming and stress responses, ultimately manifesting as physiological costs such as increased oxygen consumption, LD accumulation, and elevated COR levels. As flow velocity increases, the fish exhibit a pronounced tendency to drift backward, necessitating faster swimming speeds to maintain station. This requires greater energy expenditure, which is corroborated by the rising MO_2_ and massive GLU depletion observed in this study. Furthermore, elevated LD content may induce fatigue in fishes, while concurrent increases in COR levels trigger distinct stress responses [39].

In the present study, increased flow velocity led to a significant increase in backward time during swimming in *L. erythropterus*, which may be attributed to locomotor fatigue and stress responses. Notably, at a moderate flow condition that elicited relatively limited physiological stress of 3 BL/s, although MO_2_ was higher than that in the still-water group, the increases in LD and COR levels were not severe. This indicates that despite certain hydrodynamic stimulation under this condition, the presence of sheltering structures prevented significant physiological stress, allowing the fish to maintain a healthy state of activity.

In response to the marine ecological disturbances caused by offshore wind farm development, numerous studies at home and abroad have explored the ecological impacts of offshore wind turbine foundations on fish and other vertebrate and invertebrate species [42,43,44]. Wind turbine foundations and their scour protection structures can create an artificial reef effect by modifying local flow fields, providing shelter spaces, and enhancing food availability, thereby serving as an important habitat restoration measure under the wind–fishery integration model. The results of this study further demonstrate that shelter structures with reasonable configurations can buffer high-flow stress and influence the locomotor energy consumption and physiological stress of reef-associated juvenile fish, providing experimental evidence at the behavioral–physiological level for the ecological modification of offshore wind turbine foundations and the optimization of scour protection design. However, multiple stressors induced by wind turbine foundations, including flow field alterations, noise, and electromagnetic fields, still exert combined effects on marine organisms. Relying solely on shelter structures cannot completely eliminate engineering disturbances, and future studies should integrate field in situ investigations to comprehensively evaluate the actual regulatory effects of wind turbine foundations on reef-associated fish populations under different operating conditions.

## 5. Conclusions

This study used artificial reefs as a model to explore the effects of variable flow fields on the swimming behavior and physiological status of juvenile *L. erythropterus* under sheltered conditions. The results showed that flow velocity significantly affected swimming performance and physiological metabolism in juvenile fish, and that the protective effect of artificial shelters exhibited an obvious flow effect. Moderate flow velocity (3 BL/s) positively facilitated the utilization of artificial shelters by juvenile fish. With the increase in flow velocity, the oxygen consumption rate of fish increased significantly. When the flow velocity reached 5 BL/s, the protective function of the artificial structure weakened, backward displacement of juvenile fish increased, and the levels of lactic acid and cortisol were significantly elevated. In conclusion, artificial shelter structures can provide effective flow-avoiding spaces for juvenile *L. erythropterus* only within an appropriate flow velocity range. In addition, the juvenile *L. erythropterus* used in this study were at an undifferentiated gender stage, making gender identification impossible. Potential physiological variations due to gender differences could not be excluded, which is a limitation of this study. Future studies using mature fish will help further improve the relevant mechanistic understanding.

## Data Availability

The original contributions presented in this study are included in the article. Further inquiries can be directed to the corresponding author.
